# Prevalence of morphological and structural changes in the stylohyoid chain

**DOI:** 10.4317/jced.57186

**Published:** 2020-11-01

**Authors:** Ana-Cristina-Alves Guimarães, Daniel-Humberto Pozza, Antônio-Sérgio Guimarães

**Affiliations:** 1 MSc in Orofacial Pain and TMD, Faculdade São Leopoldo Mandic, Rua Dr. José Rocha Junqueira, 13 - 13045-755 – Campinas, Brazil; 2 DDS, PhD, Associate Professor, Departamento de Biomedicina da Faculdade de Medicina, and Faculdade de Ciências da Nutrição e Alimentação, and I3s, Universidade do Porto, Porto, Portugal; 3 DDS, PhD, Faculdade São Leopoldo Mandic, Rua Dr. José Rocha Junqueira, 13 - 13045-755 – Campinas, Brazil

## Abstract

**Background:**

Total or partial calcification of the stylohyoid chain, elongation of the stylohyoid process of the temporal bone and/or calcification of the stylohyoid ligament are usually incidental radiographic findings. The purpose of this study was to evaluate the prevalence of morphological and structural changes within the stylohyoid chain in 4413 digital panoramic radiographs.

**Material and Methods:**

The images were evaluated for the presence or absence of changes in the stylohyoid chain by a specialist in dentofacial radiology and the information collected comprised gender, age, side, right and left measurements and classification of the chain side elongated or calcified stylohyoid process, as well as type and pattern of right or left calcification.

**Results:**

275 (6.2%) subjects presented alterations, mostly bilateral, in the stylohyoid chain, being 186 females (67.6%) and 89 males (32.4%), with a higher proportion elongation. Partial calcification was more prevalent than total calcification. The right side was most affected and the frequency of events increased with age.

**Conclusions:**

The findings of the present study demonstrate that the commonest alterations in the stylohyoid chain are bilateral, mainly elongation, with a clear trend to increased prevalence with increasing age, presenting a low incidence. Noneless, the clinicians must be aware of these alterations in the routinely radiographic examination.

** Key words:**Stylohyoid process, Stylohyoid ligament calcification, Stylohyoid chain, Eagle’s syndrome.

## Introduction

A syndrome associated with alterations within the stylohyoid chain that lead to severe symptoms of atypical facial pain was first described by Eagle in 1937 and published in 1948 (1). Later on, the Eagle Syndrome was divided into classic and carotid artery syndrome, due to different symptoms associated with different anatomical locations (2). The normal length of the stylohyoid process ranges from 2.5 to 3.0 cm and elongation of this process may cause Eagle Syndrome. When the alteration is more severe than a simple elongation of the stylohyoid process, the sensation of a foreign body in the throat and a palpable hard mass in the tonsillar region should be present. Other symptoms described for this syndrome include: dysphagia, odynophagia, persistent sore throat, neck pain when rotating the head, pain when moving the tongue, cough, voice change, facial pain, otalgia, nonspecific headache, tinnitus, increased salivation, vertigo, nose pain, high neck pain, trismus, neurological and vascular problems. Thus, clinical examination and even local anaesthesia in the tonsillar fossa should be part of the diagnostic process (3-11). Among other possible etiological factors for the elongation of the stylohyoid process and / or mineralization of the stylohyoid ligament are the whiplash injury, as well as reactive hyperplasia, reactive metaplasia and anatomical variation (3,4,12-15).

Calcification of the stylohyoid ligament may be associated with an autosomal dominant background, affecting between 4 and 28% of the population (16). Eagle Syndrome is, in many cases, a condition acquired after tonsillectomy or trauma to the neck, being symptomatic in only 4% of cases (17). The reported prevalence of Eagle Syndrome is variable in the literature, ranging between 0.4 and 84.4% (18). In relation to age, the incidence varies from 45.3% for the first decade of life, 86.2% for the third, with an increase in the fourth and seventh decades, representing 87.3% and 92.0%, respectively; peaking at the eighth decade with 100% (19). There is difference between the genders for the development of the Eagle Syndrome (20). On the other hand, a higher incidence in females was found, when compared to males, in the 50-59-year age group, which continued to increase with age, especially in individuals diagnosed with systemic diseases (21).

In order to facilitate diagnosis, some authors have proposed different classifications such as into types I to III (I – elongated, II – pseudoarticulated, III - segmented) and based on degrees of ossification (A – outline, B – partial, C – nodular, D – complete) (18,22). Despite the low incidence, it is very important to recognize the symptoms of Eagle Syndrome for the differential diagnosis with other diseases (10), which justifies the need for further studies in this area. Therefore, the objectives of this study were to evaluate the prevalence of the stylohyoid chain calcification in digital panoramic radiographs regarding the following variables: age, gender, side, type, pattern and size of calcification.

## Material and Methods

The research project was approved by the Ethics and Research Committee of the São Leopoldo Mandic Dental Research Center, protocol number 1336094. The inclusion criteria comprised digital panoramic radiographs performed at the same accredited Radiology Center. The exclusion criteria included digital panoramic radiographs which did not allow clear visualization of the stylohyoid chain. Thus, a total of 4413 digital panoramic radiographs, taken between 2005 and 2014, which were in accordance with the inclusion / exclusion criteria, were selected from a database of 5683 orthodontic treatment planning file from a private clinic. The images were evaluated for the presence or absence of changes in the stylohyoid chain by a specialist in dentofacial radiology on three separate occasions to ensure accuracy. Kappa test was performed to ensure very high concordance.

Changes in the stylohyoid chain were found in 275 digital panoramic radiographs (6.2%), which were the object of this study. The following information was entered in a Table: gender, age, side, right and left measurements and classification of the chain side elongated or calcified stylohyoid process, as well as type and pattern of right or left calcification. Measurements were performed with a calliper following the lower base reference of the external acoustic meatus as the upper and lower references of the stylohyoid process or extension of the stylohyoid chain.

The ages of the volunteers and measurements of the stylohyoid chains were initially analysed using the Bartlett and Kolmogorov & Smirnov tests to observe, respectively, equality of variance and the distribution of the data. As there was no normal distribution or equality of variance, the data were analysed by means of non-parametric statistics. The ages between the genders were compared using the Mann-Whitney test, which was also used to analyse the measurements of the right and left stylohyoid chains for unilateral changes. The Wilcoxon test was used in the bilateral alterations. The comparison between bilateral and unilateral was performed with the Kruskal-Wallis test. This test was also used to observe the influence of gender and age on stylohyoid findings. The Spearman correlation test was used to study the correlation between age and measurements of the stylohyoid chain. Comparative categorical variables on distributions were analysed using the Chi-Square test, where applicable. The level of significance was 95% (α = 0.05) and the statistical software used were Bio Estat 5.0 and Graph Pad Prism version 6.0. The results obtained, as well as their variables, were demonstrated and analysed based on Tables and charts.

## Results

From the 275 digital panoramic radiographs that presented morphological alterations in the stylohyoid chain, 186 were females (67.6%) and 89 males (32.4%, *p* <0.0001). The mean age difference between women (29.2 ± 0.73 years) and men (24.6 ± 1.0 years) was significant (*p* = 0.0003). In relation to the stylohyoid chain alterations, it was verified that 153 (55.6%) were asymmetric, 20 (7.3%) were symmetric, 58 (21.1%) unilateral for the right side and 44 (16.0%) unilateral for the left side.  Table 1 shows the relative distribution of some characteristics of the sample as a function of the side (right, left or bilateral).

**Table 1:**
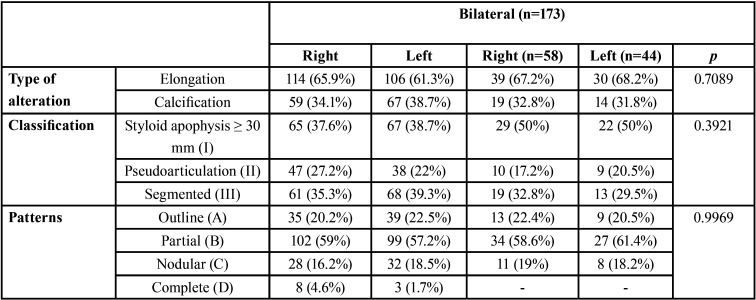
Relative distribution of the sample as a function of the side on which the changes were detected.

-Effect of gender on results

Side of the alteration (*p*=0.7164), symmetry (*p*=0.9435), type of alteration right side (*p*=0.1493), type of alteration left side (*p*=0.7393), type of alteration in right side according Langlais classification (*p*=0.0856), type of alteration in left side according Langlais classification (*p*=0.7766) presented no differences. However, there were significant differences between the genders in the sample when patterns of calcification were analysed, as shown in  Table 2.

**Table 2:**
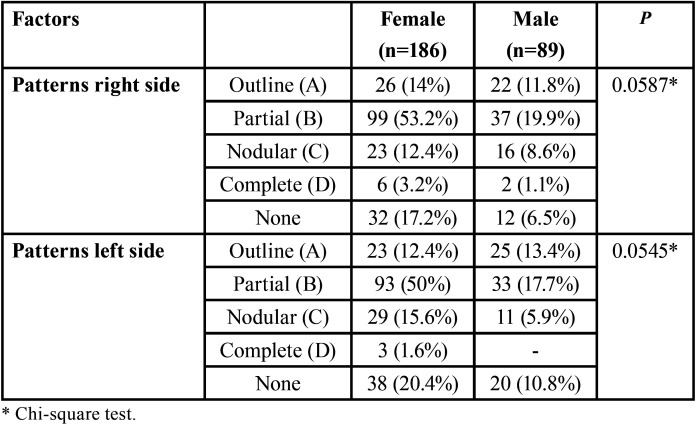
Relative distribution of the calcification patterns under study according to gender.

-Effect of age on results

 Table 3 shows the results obtained according to age group, classified according to the World Health Organization (Child - 0 to 9 years old, Adolescent 10 to 14 years old, 15-24 years old, Adult - 25 to 59 years and Elderly - 60 or more). The age groups were divided into two categories due to the low numbers.

**Table 3:**
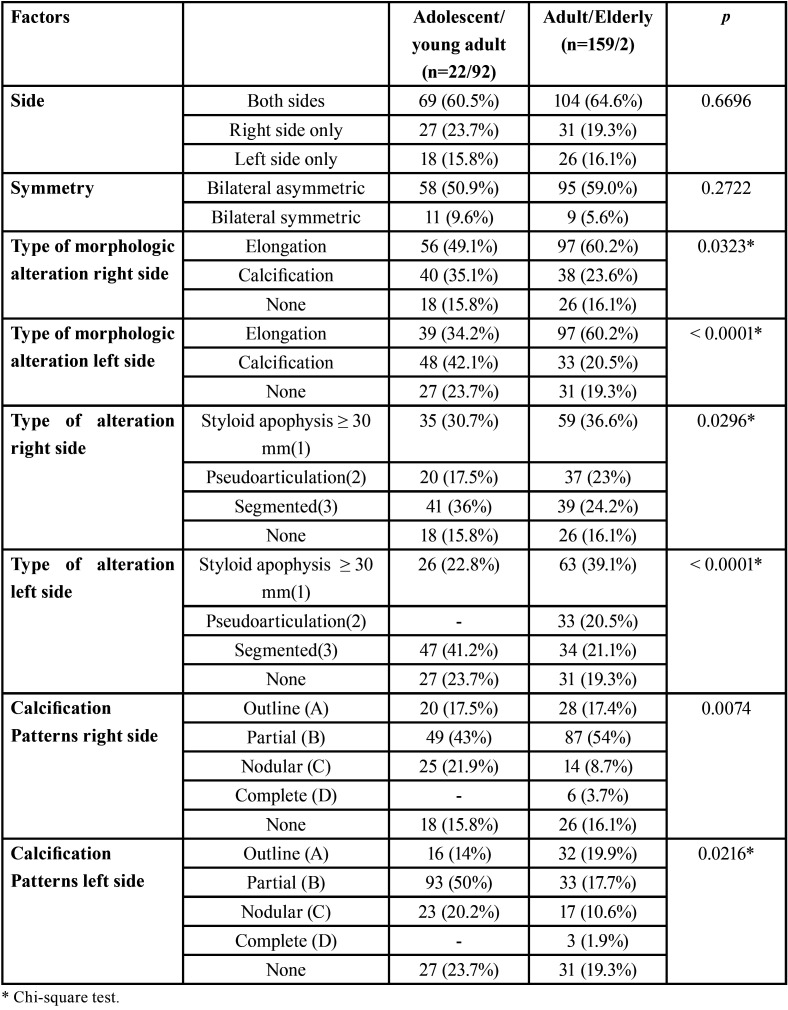
Distribution of the sample according to age groups and other factors.

## Discussion

The findings of this study demonstrated that only a small percentage of the sample population presented alterations in the stylohyoid chains. Elongation of the stylohyoid process and calcification of the stylohyoid ligament are common events and more prevalent bilaterally than unilaterally (5,20,22-26). In the present study, there were no significant differences between right and left side, for both unilateral and bilateral calcifications. The distribution of these variables was therefore considered homogeneous.

The most prevalent alteration of the stylohyoid chain was elongation, with values almost twice as high as calcification. Regarding the former, no difference in size was observed between left and right sides. However, the size of the stylohyoid chain was significantly smaller in those who presented with calcified ligaments in relation to the elongated ligaments. The clinical importance of checking for elongation of the stylohyoid process lies in the increased risk of Eagle Syndrome, especially in the case of calcification (4,6-11,22). Only a very small number of individuals with an elongated styloid process, approximately 4%, however, are symptomatic and in patients with a bilaterally elongated stylohyoid process, the symptoms are mostly unilateral (24).

According to the classification of the types and patterns of calcification, a greater proportion of type I stylohyoid process was observed. This finding is in accordance with that reported by Eagle, who classified the stylohyoid process as normal when it did not exceed 3 cm in length. With advances in imaging tests, more detailed analyses can be achieved, including calcification patterns. In the case of the present study, partial calcification was the commonest. Complete calcification was the least prevalent, probably due to longer development time involved and the symptoms most commonly reported in the latter (3,4,6-11,22). It should also be considered that, in many cases, such changes in the stylohyoid process are associated with important clinical symptoms and may require surgical intervention for patient comfort (7). Therefore, many of the significant changes may not appear in studies such as this, since the stylohyoid process would have already been removed.

In this study, and as expected, there was a tendency to increased prevalence of alterations with increasing age. Although the sample was representative, age group division was only two-fold, namely adolescents/young adults and adults/elderly, to facilitate comparisons. There was a higher proportion of calcification of the stylohyoid ligament in adolescents/young adults than in the adults/elderly group, on both sides. There was also a higher proportion of segmented calcifications on the right side in adolescents/young adults. On the left side, pseudo-articulated type alterations were most prevalent in adults/elderly. There was no influence of age in the size of the stylohyoid process. However, for some subgroups, based on classification and pattern, significant differences were observed between the right and left sides when comparing adolescents/young adults and adults/elderly. There was a greater prevalence of stylohyoid elongation as age increased and decrease in the occurrence of calcification of the stylohyoid ligament as age increased, which corroborates previous studies (19,21). In addition, is known that the highest prevalence of Eagle Syndrome occurs in the 40-50 age group (26).

Regarding gender, despite a higher number of females than males, no significant differences between genders were observed for any of the variables studied, which is also in agreement with several previous studies (20,27). However, many authors found a greater prevalence in females, approximately 3:1 in relation to men (21,24,26,28), which is higher than the values presented herein, approximately 2:1.

Most of the authors reviewed in this study reported their findings on film-based panoramic radiographs. Such radiographic examination is adequate since images from the stylohyoid chain should not be duplicated. The duplication of images usually occurs in the midline where structures are intercepted twice by the X-ray beam, not affecting the lateral stylohyoid images. The advantages of using digital panoramic radiographs, instead of film-based, are mainly related to storage, enhanced communication and the possibility of enhancing desired areas of interest (20,21,26,29). Regardless the methodology, the results in the literature are very heterogeneous since some authors (30), evaluating a 100 panoramic radiographs, reported only 1% of alterations, while others (31) evaluating 286 panoramic radiographs found much more, 84.4%.

Finally, considering the limitations of a retrospective study, it was not possible to collect data on prevalence of clinical signs and symptoms associated with Eagle Syndrome or even temporomandibular disorders. In conclusion, the findings of the present study demonstrate that the commonest alterations in the stylohyoid chain are of low incidence, bilateral, mainly elongation, with a clear trend to increased prevalence with increasing age. Noneless, the clinicians must be aware of these alterations in the routinely radiographic examination.
